# Virtual Reality for Pain, Anxiety, and Patient Experience in Awake Urological Procedures: A Systematic Review and Meta-Analysis of Randomised Controlled Trials

**DOI:** 10.7759/cureus.117838

**Published:** 2026-10-13

**Authors:** Alhassen Alseneid, Ali Alseneid, Shivam Bhindi, Thanmay Mangala Sachidananda, Haiderah Alseneid, Ola Blach

**Affiliations:** 1 Medicine, East Kent Hospitals University NHS Foundation Trust, Canterbury, GBR; 2 Medicine, William Harvey Hospital, Ashford, GBR; 3 Medicine, King's College London GKT School of Medical Education, London, GBR; 4 Pharmacology, Kingston University, London, GBR; 5 Urology, Princess Royal Hospital, Haywards Heath, GBR

**Keywords:** anxiety, cystoscopy, non-pharmacological intervention, pain management, patient experience, prostate biopsy, systematic review, virtual reality

## Abstract

Awake urological procedures, including cystoscopy, transrectal ultrasound-guided prostate biopsy (TRUS-Bx), extracorporeal shock wave lithotripsy, vasectomy, and urodynamic studies, are consistently associated with procedural pain and anxiety, with downstream consequences for surveillance compliance and patient experience. Head-mounted display (HMD) virtual reality (VR) has emerged as a non-pharmacological adjunct, but existing reviews have been narrative or restricted to a single procedure type. This systematic review and meta-analysis evaluated head-mounted display VR across the full breadth of awake urological procedures.

PubMed, Embase, and the Cochrane Central Register of Controlled Trials were searched from inception to April 2026. Eligible studies were randomised controlled trials (RCTs) of HMD VR compared with standard care in adults undergoing office-based urological procedures under local anaesthesia. Two reviewers independently performed study selection, data extraction, and risk of bias assessment using the revised Cochrane risk of bias tool (RoB 2). Random-effects meta-analyses used the standardised mean difference (SMD) with Hedges' g. Certainty of evidence was rated using the Grading of Recommendations, Assessment, Development and Evaluations (GRADE) framework. Reporting followed the Preferred Reporting Items for Systematic Reviews and Meta-Analyses (PRISMA) 2020 statement.

Eleven RCTs encompassing 1,557 participants across eight countries were included. The VR was associated with statistically significant reductions in procedural pain (SMD −0.68, 95% CI −1.25 to −0.11) and anxiety (SMD −1.39, 95% CI −2.64 to −0.14), although both estimates carried substantial heterogeneity (*I*² = 91.7% and 89%, respectively). Subgroup analysis of pain by procedure type revealed marked divergence: VR produced negligible benefit during cystoscopy (SMD −0.16, 95% CI −0.32 to 0.00) but a large reduction during TRUS-Bx (SMD −1.84, 95% CI −2.82 to −0.85), the latter based on only two studies with substantial heterogeneity. Narrative synthesis showed consistent improvements in patient satisfaction and procedural comfort and attenuation of physiological stress markers. No serious VR-related adverse events were identified across approximately 900 patients. The certainty of evidence was low for all outcomes.

The HMD VR may offer meaningful reductions in procedural pain and anxiety during selected awake urological procedures, particularly those characterised by sustained nociceptive stimulation and high anticipatory distress, with a consistent safety profile. However, the very high between-study heterogeneity means these pooled averages should be interpreted cautiously, and the benefit appears procedure-specific rather than uniform. The benefit appears procedure-specific and is unlikely to exceed established pharmacological standards during routine cystoscopy. Adequately powered procedure-specific trials are needed before VR can be considered a standard adjunct.

## Introduction and background

Cystoscopy, transrectal ultrasound-guided prostate biopsy (TRUS-Bx), extracorporeal shock wave lithotripsy (ESWL), vasectomy, and urodynamic studies are among the most frequently performed urological investigations and therapeutic interventions, the majority of which are conducted in the awake outpatient setting under local anaesthesia. More than two million prostate biopsies are performed annually in the United States and Europe alone, and despite their routine nature, these procedures are consistently associated with significant patient-reported discomfort [1]. Procedure-related distress is common: approximately 49% of men undergoing prostate biopsy experience clinically significant anxiety [2]. Procedure-related distress has in turn been associated with reluctance to undergo repeat biopsy, which may contribute to under-sampling and delayed diagnosis, although the strength of this association varies across studies [2]. Non-pharmacological strategies have been explored to mitigate this distress, including brief mind-body interventions during biopsy [3], motivating interest in immersive alternatives such as virtual reality. In cystoscopy, pre-procedural anxiety has been shown to independently account for up to 38.6% of the variance in intraoperative pain scores, and anxiety and depression are well-documented in patients undergoing diagnostic cystoscopy [4,5]. Inadequately managed procedural anxiety and pain are associated with reduced compliance with surveillance schedules, impaired physician-patient communication, and increased sympathetic stress responses, all of which may compromise both diagnostic quality and procedural safety.

Virtual reality (VR) is an immersive, computer-generated technology that uses head-mounted displays (HMDs) to envelope the user in a three-dimensional audiovisual environment. Its analgesic and anxiolytic effects are attributed to competition for finite attentional and sensory processing capacity, limiting the conscious perception of pain and anxiety [6-8]. Clinical evidence supports VR's effectiveness across burn wound debridement, paediatric venepuncture, dental procedures, gastrointestinal endoscopy, and percutaneous cardiovascular interventions, with a consistently favourable safety profile dominated by mild and transient nausea or dizziness [8,9].

Within urology, individual randomised controlled trials (RCTs) have evaluated VR across cystoscopy, TRUS-Bx, ESWL, and urodynamic studies. A previous narrative mini-review summarised this emerging field but did not undertake a formal quantitative synthesis [10]. A 2025 systematic review and meta-analysis subsequently provided the first quantitative urological synthesis but was restricted to cystoscopy [11]. No synthesis has yet quantified VR across the full range of awake urological procedures or formally appraised the certainty of the underlying evidence. The present systematic review and meta-analysis was therefore conducted to synthesise the available randomised controlled trial evidence examining HMD-based VR as a non-pharmacological adjunct across the full range of awake urological procedures, including cystoscopy, TRUS-Bx, ESWL, vasectomy, and urodynamic studies. The primary outcomes were procedural pain and procedural anxiety; secondary outcomes included patient satisfaction and procedural comfort, physiological markers of stress, and procedure-related adverse events. Risk of bias was assessed using the revised Cochrane risk of bias tool (RoB 2) [12], and the certainty of evidence was evaluated using the Grading of Recommendations, Assessment, Development and Evaluations (GRADE) framework [13].

## Review

Materials & Methods

Protocol and Registration

This systematic review and meta-analysis was conducted and reported in accordance with the Preferred Reporting Items for Systematic Reviews and Meta-Analyses (PRISMA 2020) statement [14]. The review protocol is registered on the International Prospective Register of Systematic Reviews (PROSPERO) (registration number: CRD420261379628).

Eligibility Criteria

Studies were eligible if they were RCTs of adult patients (≥18 years) undergoing an office-based urological procedure under local anaesthesia or minimal sedation, in which a HMD VR device was compared with standard care or an equivalent control condition, and at least one eligible outcome was reported. Eligible procedures included flexible and rigid cystoscopy, TRUS-Bx, transperineal prostate biopsy, ESWL, vasectomy, urodynamic studies, and ureteric stent removal. Eligible outcomes were procedural pain (visual analogue scale (VAS) or numerical rating scale (NRS)), procedural anxiety (validated instruments including the State-Trait Anxiety Inventory (STAI) [15] or VAS-anxiety [16]), patient satisfaction or procedural comfort, physiological markers (heart rate (HR), blood pressure (BP), peripheral oxygen saturation (SpO₂)), and procedure-related adverse events. Studies were excluded if they used non-HMD VR systems, involved general or regional anaesthesia, were non-randomised, exclusively paediatric, or were conference abstracts without sufficient extractable data. No language restrictions were applied. The full inclusion and exclusion criteria are summarised in Table 1.

**Table 1:** Inclusion and exclusion criteria RCTs: Randomised controlled trials; HMD: Head-mounted display; VR: Virtual reality; TRUS-Bx: Transrectal ultrasound-guided prostate biopsy; ESWL: Extracorporeal shock wave lithotripsy; VAS: Visual analogue scale; NRS: Numerical rating scale; STAI: State-Trait Anxiety Inventory

Parameter	Inclusion criteria	Exclusion criteria
Study design	RCTs	Non-randomised or retrospective designs; conference abstracts without sufficient extractable data
Participants	Adults aged ≥18 years	Exclusively paediatric populations
Setting	Office-based (outpatient) urological procedure	Non-office-based settings
Anaesthesia/sedation	Local anaesthesia or minimal sedation	General or regional anaesthesia
Intervention	HMD VR	Non-HMD VR systems
Comparator	Standard care or an equivalent control condition	—
Procedures	Flexible or rigid cystoscopy; TRUS-Bx; transperineal prostate biopsy; ESWL; vasectomy; urodynamic studies; ureteric stent removal	Any procedure outside the eligible list
Outcomes (≥1 required)	Procedural pain (VAS or NRS); procedural anxiety (validated instruments, e.g. STAI or VAS-anxiety); patient satisfaction or procedural comfort; physiological markers (heart rate, blood pressure, oxygen saturation); procedure-related adverse events	No eligible outcome reported
Language	No language restriction	—

Information Sources and Search Strategy

PubMed, Embase, and the Cochrane Central Register of Controlled Trials (CENTRAL) were systematically searched from inception to April 2026. Search terms combined controlled vocabulary (Medical Subject Headings (MeSH) and Emtree terms) with free-text keywords relating to virtual reality, head-mounted displays, and the eligible urological procedures. Reference lists of all included studies and relevant systematic reviews were screened manually. The search strategy is summarised in Table 2.

**Table 2:** Search strategy Total records identified = 243. MeSH: Medical Subject Headings; VR: Virtual reality; HMD: Head-mounted display; TRUS: Transrectal ultrasound; ESWL: Extracorporeal shock wave lithotripsy

Database (platform)	Vocabulary	Search concepts/terms	Date range	Records
PubMed (NLM)	MeSH + free text	Concept 1 (VR): “virtual reality” OR “head-mounted display” OR HMD OR immersive OR “virtual environment”. Concept 2 (procedure): cystoscopy OR “prostate biopsy” OR “transrectal ultrasound” OR TRUS OR “shock wave lithotripsy” OR ESWL OR vasectomy OR urodynamic OR “ureteric stent”. Combined: Concept 1 AND Concept 2.	Inception – April 2026	42
Embase (Ovid)	Emtree + free text	As above (adapted to Emtree syntax).	Inception – April 2026	118
Cochrane CENTRAL (Cochrane Library)	MeSH + free text	As above (adapted to CENTRAL syntax)	Inception – April 2026	83
Hand-searching	—	Reference lists of all included studies and relevant systematic reviews.	—	Not separately quantified

Study Selection

Records were imported into Rayyan systematic review software (Rayyan Systems Inc., Cambridge, MA, USA) for deduplication and screening. Two reviewers (AA1, AA2) independently screened titles and abstracts and assessed full-text eligibility, with disagreements resolved by consensus or arbitration with remaining authors. The selection process is reported using a PRISMA 2020 flow diagram.

Data Collection and Data Items

Data were extracted independently by the same two reviewers using a pre-specified standardised form. Extracted data comprised study citation, country, design, randomisation method, procedure type, sample size, demographics, anaesthesia and analgesia type, VR device, content type (passive or active/interactive), delivery timing, comparator, outcome data (means, standard deviations, p-values), adverse events, and dropout rates. Where standard deviations were not reported directly, they were derived from 95% confidence intervals or standard errors using standard Cochrane formulae. For Walker et al. [17], standard deviations were unavailable in the primary report and were obtained from the prior systematic review by Lopes et al. [11].

Outcomes

The primary outcomes were procedural pain and procedural anxiety. Secondary outcomes were patient satisfaction or procedural comfort, physiological markers of stress (heart rate, blood pressure, oxygen saturation), and procedure-related adverse events. Where multiple instruments measured the same construct, validated instruments were prioritised, and pain scores were analysed as standardised mean differences (SMDs) to accommodate different scales.

Risk of Bias (RoB)

The RoB was assessed independently by two reviewers using the RoB 2 tool, evaluating five domains and assigning an overall judgement of low risk, some concerns, or high risk [12]. Disagreements were resolved by discussion. Blinding of participants and care providers was not feasible in any included trial given the nature of VR interventions; this was acknowledged as a systematic limitation of the evidence base rather than a study-specific failing.

Statistical Analysis

Quantitative synthesis was performed where studies reported sufficient data for the same outcome using comparable instruments. The SMD, Hedges' g with 95% CIs, was used as the summary measure for all continuous outcomes. A negative SMD indicates a lower score in the VR group, favouring VR for pain and anxiety. All pooled analyses used a random-effects model with the DerSimonian and Laird method [18]. For the anxiety outcome (k = 4), the Knapp-Hartung adjustment was applied to provide more conservative confidence intervals, as recommended for meta-analyses with few studies [19]. For pain (k = 7), the standard DerSimonian-Laird model was used.

Statistical heterogeneity was assessed using the Cochran's Q test and the I² statistic, interpreted in accordance with Cochrane Handbook guidance [20]. Formal assessment of publication bias was not performed, as funnel plots and regression-based tests are underpowered and unreliable with fewer than 10 studies per outcome [19]. Subgroup analyses were pre-specified by procedure type (cystoscopy, TRUS-Bx, ESWL). For the anxiety outcome, subgroup forest plots were not generated due to the small number of contributing studies per subgroup; procedure-specific results are reported narratively. Outcomes for which quantitative pooling was not feasible, patient satisfaction and comfort, physiological markers, and adverse events, were synthesised narratively. All statistical analyses were performed using Meta-Analysis Online (www.metaanalysisonline.com), with statistical significance defined as P < 0.05.

Certainty of Evidence

The certainty of evidence for each outcome was assessed using the GRADE framework, with downgrading considered for risk of bias, inconsistency, indirectness, imprecision, and publication bias. Overall certainty was classified as high, moderate, low, or very low [13].

Results

The systematic search identified 243 records (PubMed n = 42; Embase n = 118; Cochrane n = 83). Following the removal of 65 duplicates, 178 records were screened at the title and abstract level, of which 101 were excluded. Seventy-seven reports were sought for retrieval, of which 55 could not be retrieved. The remaining 22 reports were assessed for full-text eligibility. Of these, 11 were excluded (conference abstracts without extractable data n = 4; ongoing trials without published results n = 5; general anaesthetic setting n = 1; retrospective design n = 1). Eleven RCTs meeting all criteria were included [17,21-30]. The selection process is summarised in the PRISMA 2020 flow diagram (Figure 1).

**Figure 1:**
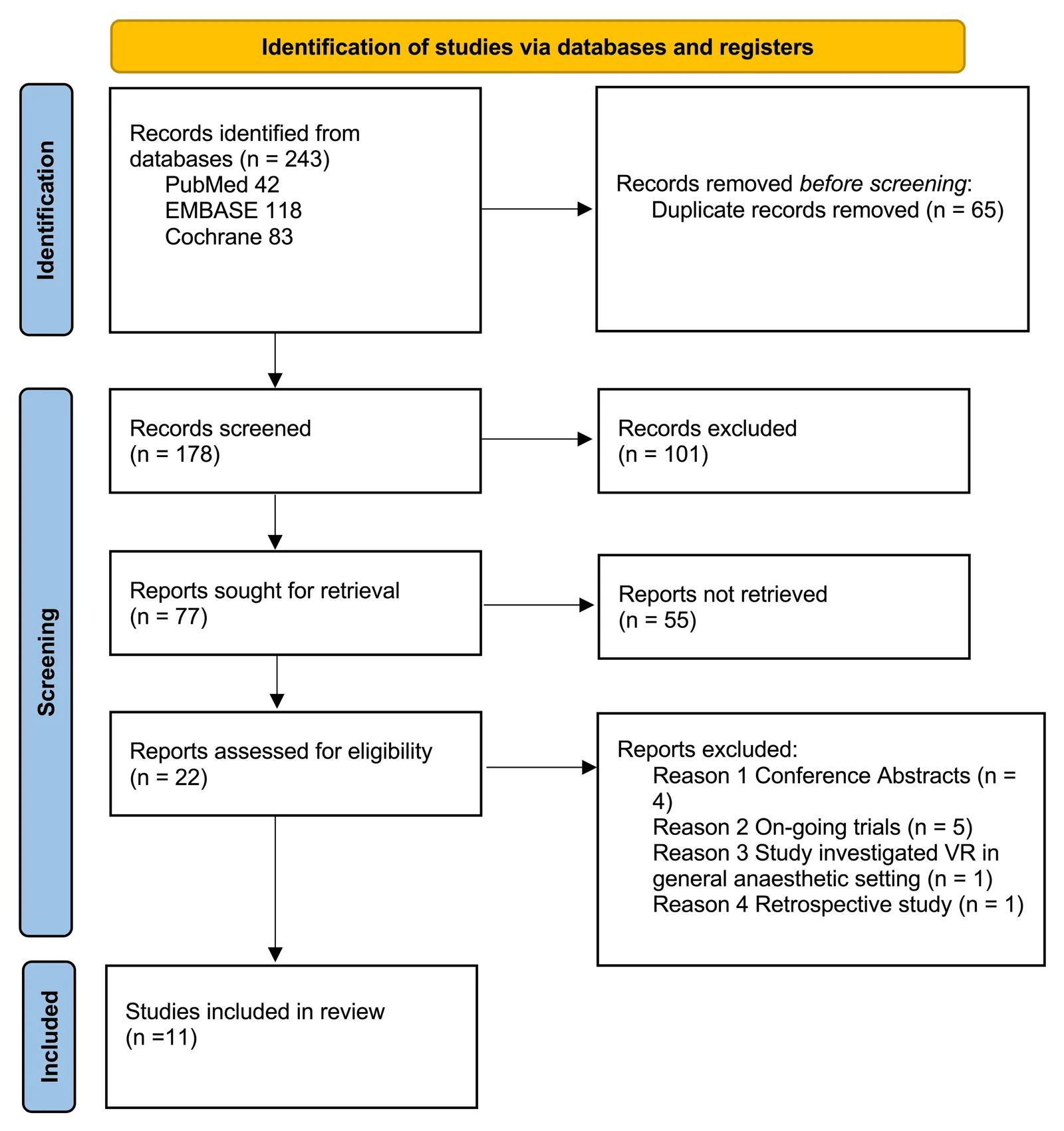
PRISMA 2020 flow diagram of study identification, screening, and inclusion PRISMA: Preferred Reporting Items for Systematic Reviews and Meta-Analyses

Study Characteristics

The 11 included RCTs were published between 2014 and 2026 and collectively enrolled 1,557 participants across eight countries (Turkey n = 4, Brazil n = 1, Thailand n = 1, Poland n = 1, Belgium n = 1, Canada n = 1, New Zealand n = 1, USA = 1). Procedures included rigid cystoscopy (n = 2), flexible cystoscopy (n = 2), TRUS-Bx (n = 5), ESWL (n = 1), vasectomy and flexible cystoscopy combined (n = 1), and urodynamic studies (n = 1). Sample sizes ranged from 42 to 270 participants. Mean age ranged from 37.5 to 73.1 years; ten of eleven studies enrolled exclusively male participants, with Sibal et al. the only study with substantial female representation [28]. All studies delivered VR via HMD, using passive immersive content in eight studies and active or interactive content in three [17,24,27]. The VR was delivered intraprocedurally in 10 studies and perioperatively in one [24]. All studies used standard care as a comparator; one included an enhanced control with monitor viewing and physician interaction [17]. Full study characteristics are presented in Table 3.

**Table 3:** Study characteristics BP: Blood pressure; DBP: Diastolic blood pressure; EMLA: Eutectic mixture of local anaesthetics; ESWL: Extracorporeal shock wave lithotripsy; FLACC: Face, legs, activity, cry, consolability scale; HMD: Head-mounted display; HR: Heart rate; ITT: Intention-to-treat; NRS: Numerical rating scale; PPHEN: Patient perception of hospital experience with nursing scale; PROMIS: Patient-reported outcomes measurement information system; RCT: Randomised controlled trial; SBP: Systolic blood pressure; SpO₂: Peripheral oxygen saturation; STAI: State-Trait Anxiety Inventory; TAU: Treatment as usual; TRUS-Bx: Transrectal ultrasound-guided prostate biopsy; VAS: Visual analogue scale; VR: Virtual reality

Author & year of study	Procedure type	Study design	Total n (VR/control)	Mean age (years)	Sex (% male)	Anaesthesia/analgesia	VR device	VR content type	VR delivery timing	Comparator	Outcomes measured
Goergen & Freitas, 2022 [24]	Rigid cystoscopy	Prospective RCT (block randomisation by day)	n=159 (VR=80 / control=79)	VR: 62.1±13.4, control: 65.1±12.0	67.3%	2% intraurethral lidocaine gel (20 mL, two minutes pre-procedure)	Samsung A5 phone + Trust Urban Exos 3D VR glasses + headset	Passive - rail-ride app video. Intraprocedural distraction.	Intraprocedural	Standard care (no VR)	Pain (VAS), discomfort (VAS), HR variation, procedure duration, difficulty (VAS)
Ketsuwan et al., 2022 [25]	Flexible cystoscopy	Prospective RCT, single-centre, open-label (computer block randomisation; sealed envelopes)	n=270 (VR=135 / control=135)	VR: 64.4±12.2, control: 65.4±11.2	63.3%	10 mL 2% intraurethral lidocaine gel; Olympus CYF-VHA flexible cystoscope	BOBOVR Z6 Bluetooth HMD + Apple iPhone 12	Passive - static/ dynamic snowy mountain landscape; relaxing background music.	Pre- and intraprocedural (10 minutes before through end)	Standard care (no VR device)	Anxiety (STAI-SA), pain (VAS), satisfaction (VAS), willingness to repeat (VAS), HR, SBP, DBP, SpO2
Łuczak et al., 2021 [26]	Rigid cystoscopy	Prospective RCT (computer-generated; patient blinded to allocation)	n=103 (VR=52/control=51)	VR: 65.6±11.4, control: 67.3±11.1	71.8%	2% lidocaine lubricating gel; rigid 20 French cystourethroscope; no systemic sedation	VR goggles + headphones (brand not named; specialist-selected content)	Passive - Skogafoss waterfall image; dynamic elements; head-tracking; nature soundscape.	Intraprocedural	Standard care (no VR set)	Pain (NRS), fear (NRS), nausea (NRS), FLACC (non-verbal pain), BP, HR, SpO2
Walker et al., 2014 [17]	Flexible cystoscopy	Prospective RCT (random number generator; single urologist)	n=45 (VR=22/control=23; 43 analysed)	VR: 38, control: 47 (not stated)	100% (males only)	2% intraurethral lidocaine jelly; control could view the procedure on a monitor	VR helmet + trackball hand controller (SnowWorld environment)	Active/interactive - SnowWorld game (shoot snowballs at targets). Low immersion reported (22/100 mm).	Intraprocedural	Standard care + monitor viewing + physician interaction	Pain (VAS), anxiety (VAS), time thinking about pain (VAS), vital signs, galvanic skin response
Genç et al., 2022 [22]	TRUS prostate biopsy	3-arm RCT (VR / stress ball/control; block randomisation) (VR vs control extracted)	n=96 (VR=32/stress ball=32/control=32)	VR: 70.6±7.9, control: 73.1±8.2	100%	Standard protocol: lidocaine gel or short-acting narcotics (type not specified)	Smartphone + VR HMD (brand not named)	Passive - 360° nature video scenes from YouTube; 10 minutes duration.	Intraprocedural (~10 minutes; matching biopsy duration)	Standard care (no intervention)	Pain (VAS; recall 15 minutes post-procedure), HR, systolic blood pressure (SBP), diastolic blood pressure (DBP), SpO2, respiratory rate (no anxiety scale)
Toraman & Ilgin, 2024 [29]	TRUS prostate biopsy	RCT, pre/post-test (two-block randomisation via random.org; single surgeon)	n=70 (VR=35/control=35)	VR: 64.1±5.3, control: 64.3±4.5	100%	Standard care (analgesia type not specified; periprostatic nerve block implied)	VR glasses (brand not named; 360° YouTube content)	Passive - 360° YouTube videos: nature, parks, sea, museum tours; background music; 10- 15 minutes.	Intraprocedural (VR applied two minutes before start; worn until procedure end)	Standard care (routine nursing care)	Pain (VAS; 10 minutes post- procedure), anxiety (STAI-I), trait anxiety (STAI-II), satisfaction (patient perception of hospital experience with nursing scale ( PPHEN) scale)
Robinson et al., 2026 [27]	TRUS Prostate Biopsy	Single-centre parallel-group prospective RCT (block randomisation; sealed envelopes; analysts blinded) intention-to-treat (ITT) analysis	n=250 (VR=125/control=125; 18 withdrew post-randomisation)	VR: 64.1±6.7, control: 64.6±7.5	100%	Local anaesthetic per standard hospital protocol (type not specified)	Oculus Go VR headset; pre-selected immersive distraction environment	Interactive/passive hybrid - immersive distraction environment. Moderate immersion (mean 35/100).	Intraprocedural (fitted at start; worn throughout)	Standard care (no additional intervention)	Pain (VAS 0–100), anxiety (VAS subscales), satisfaction (VAS), willingness to repeat, nausea
Bozkul et al., 2026 [21]	TRUS prostate biopsy	Prospective RCT, two parallel arms (computer-generated block randomisation; sealed envelopes; data collector + analyst blinded)	n=70 (VR=35/control=35)	VR: 68.6±7.3, control: 69.8±8.3	100%	Periprostatic nerve block with bupivacaine (under TRUS; 12-core biopsy)	VR Shinecon G12 3D VR glasses + Xiaomi Redmi Note 8 Pro	Passive - Six-minute 360° nature video with relaxing music; looped to 30 minutes to cover full procedure.	Intraprocedural (applied at start; continuous loop ~30 minutes)	Standard care + routine verbal info from staff	Anxiety (STAI; primary), comfort (VAS 0–10; primary), 24-hour complications (secondary; no pain VAS)
Weynants et al., 2023 [30]	ESWL (extracorporeal shock wave lithotripsy)	Prospective RCT, parallel group (online randomizer at each session; radiologist blinded for follow-up)	n=166 (VR=82/control=84; one lost to follow-up per arm)	VR: 56.0±14.3, control: 56.8±14.6	VR: 82% control: 67% (imbalance p=0.027)	Diclofenac 100 mg suppository (1h pre) + EMLA 5g topical cream (~30 minutes pre); no sedation	Oncomfort Sedakit (Oncomfort SA, Belgium); 360° audio-visual VR hypnosis headset	Passive - immersive 3D underwater view following a whale; designed as VR hypnosis for relaxation.	Intraprocedural (after positioning; throughout 2500 shocks)	Standard care (diclofenac + EMLA only; no VR)	Pain (VAS; primary), comfort (Likert; secondary), total delivered energy (J), clinical success (stone-free)
Gentile et al., 2026 [23]	Vasectomy (n=30) + flexible cystoscopy (n=12) (analysed separately)	Feasibility pilot RCT, multi-method (open-label; computer-generated randomisation; ITT analysis) not powered for clinical outcomes	n=42 total vasectomy: VR=15/treatment as usual (TAU)=15, cystoscopy: VR=7/TAU=5	Vasectomy: 37.5±6.0, cystoscopy: 54.6±16.7 (significant difference between procedures)	100%	Vasectomy: injected lidocaine ± nitrous oxide (n=5 premium). Cystoscopy: flexible scope + lubrication jelly.	Meta Quest 3 VR headset; Tripp app (publicly available evidence-based VR meditation)	Active/interactive - mindfulness + breathing exercises + slow-moving imagery + music. Five scene options (patient-selected).	Perioperative (pre-, intra-, post-procedural) Intraop pain/anxiety at procedure midpoint	Treatment-as- usual (TAU; standard surgical care)	Feasibility metrics, pain (VAS intraop; patient-reported outcomes measurement information system (PROMIS) post-op), anxiety (VAS), distress (thermometer), satisfaction (0-10)
Sibal et al., 2025 [28]	Urodynamic studies (invasive urodynamics)	Prospective RCT, pre/post-test (computer-based research randomizer; first-time urodynamics only)	n=86 (VR=43/control=43)	VR: 48±13, control: 56±11 (significant difference p=0.004)	VR: 35%, control: 30% (mixed-sex; ~65-70% female)	No pharmacological anaesthesia or sedation. Standard urodynamic catheter protocol only.	Efnan G04EA VR Shinecon 3D headset + smartphone	Passive - pre-recorded 360° nature videos (YouTube). Audio excluded (no music/sounds) to avoid influencing urodynamic results.	Intraprocedural (full ~30-minute procedure duration)	Standard urodynamic procedure (no distraction)	Anxiety (STAI-I pre/post; primary), trait anxiety (STAI-II), patient acceptance (no pain scale)

Risk of Bias

No study was judged to have low overall RoB. Eight studies were rated with some concerns and three had high overall RoB (Figure 2). Studies rated at high risk were Genç et al., in which procedural pain was assessed by patient recall 15 minutes after procedure completion (high risk, domain 4); Robinson et al., in which 22 of 125 VR-allocated participants received standard care representing substantial crossover contamination (high risk, domain 3); and Sibal et al., in which statistically significant between-group imbalances in age (P = 0.004) and chronic disease prevalence (P = 0.005) were present despite randomisation, with a numerically higher baseline STAI-I score in the VR group raising concern for regression to the mean (high risk, domain 1) [22,27,28]. The most consistent source of bias was the inability to blind participants and care providers, an inherent and unavoidable feature of VR intervention trials. Outcome assessor blinding was reported in only one study (Bozkul et al.) [21].

**Figure 2:**
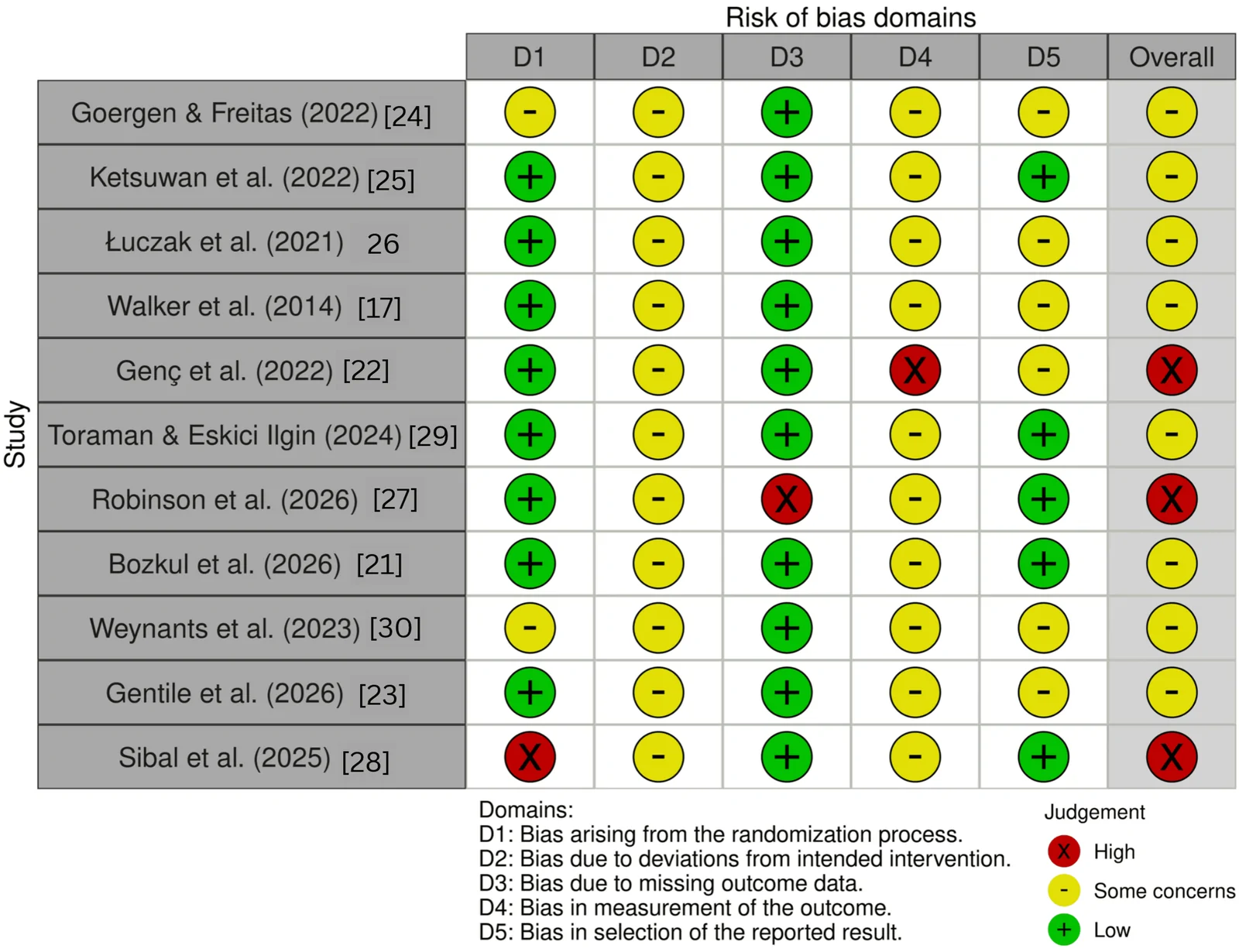
RoB 2 tool assessing risk of bias in RCT studies The RoB summary across the 11 included randomised controlled trials was assessed using the revised Cochrane RoB 2. Domains D1: Randomisation; D2: Deviations from intended interventions; D3: Missing outcome data; D4: Outcome measurement; D5: Selection of reported result [17,21-30] RoB: Risk of bias; RCT: Randomized controlled trial

Pain

Seven RCTs reporting procedural pain were identified, of which six (n = 709; VR n = 356, control n = 353) were included in the pooled meta-analysis stratified by procedure into two subgroups: cystoscopy (k = 4, n = 575) and TRUS-Bx (k = 2, n = 134). The remaining study was the sole ESWL trial and is reported descriptively [30]. Pain was assessed on a 10 cm VAS in five studies [22,24,25,29,30], a 0-10 NRS in one study [26], and a 100 mm VAS in one study [17], the latter converted to a common 0-10 scale by dividing reported values by ten. Five studies assessed pain immediately on procedure completion [17,24-26,30], while two assessed pain by patient recall after a brief post-procedural interval (Genç at 15 minutes, Toraman at 10 minutes) [22,29]. Overall, VR was associated with a statistically significant reduction in procedural pain (SMD −0.68, 95% CI −1.25 to −0.11; Z = −2.35; P = 0.019; random-effects model, Hedges' g). Heterogeneity was very high (I² = 91.7%, τ² = 0.45, χ² = 60.27, df = 5, P < 0.0001), and the 95% prediction interval (−2.56 to 1.20) crossed zero, indicating that the direction and magnitude of effect cannot be reliably anticipated in a new study population (Figure 3).

**Figure 3:**
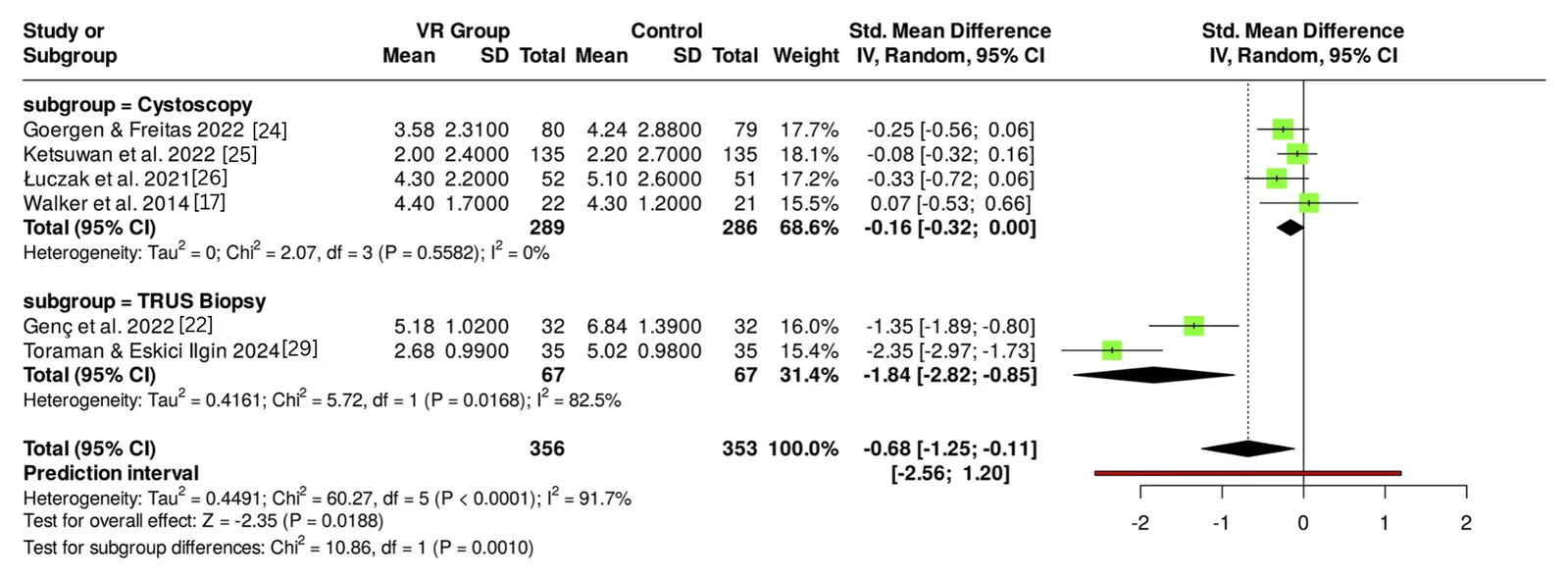
Forest plot of SMD in procedural pain stratified by procedure type (cystoscopy and TRUS-Bx) The test for subgroup differences was statistically significant (p = 0.001), demonstrating divergent effects between procedure types [17,22, 24-26,29]. TRUS-Bx: Transrectal ultrasound-guided prostate biopsy

Subgroup analysis revealed significantly divergent effects (χ² = 10.86, df = 1, P = 0.001). Among cystoscopy studies, VR produced a small, non-significant reduction in pain (SMD −0.16, 95% CI −0.32 to 0.00) with no between-study heterogeneity (I² = 0%) (Figure 3). Among TRUS-Bx studies, VR was associated with a large, statistically significant pain reduction (SMD −1.84, 95% CI −2.82 to −0.85; P = 0.017), though with substantial within-subgroup heterogeneity (I² = 82.5%); this subgroup comprised only two studies and should be interpreted cautiously. In the single ESWL study (n = 166), VR patients reported significantly lower pain scores on a 0-10 cm VAS (mean 4.01 vs 4.94; SMD −0.39, 95% CI −0.69 to −0.08), an approximately 19% reduction, and tolerated significantly higher total energy delivery (55.2 vs 48.8 J; P = 0.037), though without improvement in stone-free rates [30]. Formal assessment of publication bias was not undertaken, as funnel-plot and regression-based methods are unreliable with fewer than 10 studies.

Anxiety

Four RCTs reporting post-procedural state anxiety on the STAI were eligible for pooled analysis (VR n = 248, control n = 248) [21,25,28,29]. Random-effects meta-analysis with Knapp-Hartung adjustment demonstrated a statistically significant reduction in post-procedural anxiety favouring VR (SMD −1.39, 95% CI −2.64 to −0.14; P < 0.05; Figure 4). Heterogeneity was substantial (I² = 89%; P < 0.01). Subgroup analysis by procedure type was not performed due to the small number of contributing studies. Formal assessment of publication bias was not undertaken, as funnel-plot and regression-based methods are unreliable with fewer than 10 studies.

**Figure 4:**
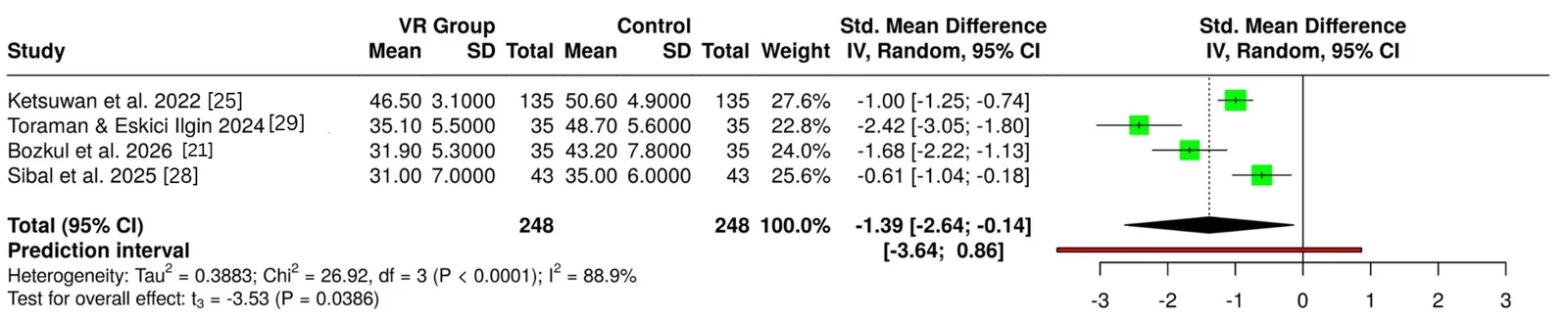
Forest plot of SMD in procedural anxiety Random-effects model with Knapp-Hartung adjustment; SMD < 0 favours virtual reality [21,25,28,29] SMD: Standardised mean difference

Seven further studies reported anxiety using non-STAI instruments, with scale heterogeneity precluding quantitative pooling. Walker et al. reported no significant between-group difference in VAS-anxiety during flexible cystoscopy [17]. Robinson et al. found all anxiety subscale comparisons non-significant, with findings likely confounded by COVID-19 facemask requirements and substantial crossover contamination [27]. Gentile et al. observed a positive trend for perioperative distress reduction in vasectomy patients without reaching statistical significance, consistent with the feasibility design [24].

Patient Satisfaction

Five RCTs reported patient satisfaction or procedural comfort. Ketsuwan et al. demonstrated significantly higher post-procedural satisfaction in the VR group during flexible cystoscopy (VAS 9.4 ± 0.9 vs 8.8 ± 1.9; P = 0.001) with greater willingness to undergo repeat procedures (2.0 ± 0.2 vs 1.8 ± 0.5; P = 0.001) [25]. Toraman and Ilgin reported significantly higher patient perception of nursing care quality following TRUS-Bx in the VR group using the validated PPHEN scale (65.4 ± 4.7 vs 42.4 ± 4.5; P < 0.001), with VR explaining 86.6% of variance in post-procedural PPHEN scores [29]. Bozkul et al. demonstrated significantly greater procedural comfort in the VR group (VAS 9.4 ± 0.7 vs 7.5 ± 1.6; P < 0.001; Cohen's d = 1.51) [20]. In contrast, Robinson et al. found no significant between-group difference in post-procedural satisfaction during TRUS-Bx (VR 79.2 vs control 77.1; mean difference (MD) 2.1, 95% CI −4.4 to 8.7; P = 0.520) or willingness to repeat (P = 0.803) [27]. Formal pooling was not performed due to heterogeneous instruments measuring related but distinct constructs.

Physiological Measures

Five RCTs reported objective physiological markers. Formal pooling was precluded by heterogeneity in metrics reported (absolute post-procedure values, peak delta from baseline, and pre-to-peri-procedural change). Heart rate was reported by four studies, all favouring VR. Goergen and Freitas reported significantly lower peak HR variation in the VR group during rigid cystoscopy (6.29 ± 6.66 vs 11.09 ± 10.17 bpm; P < 0.001) [24]. Ketsuwan et al. demonstrated significantly lower absolute post-procedural HR (87.6 ± 4.7 vs 94.1 ± 3.8 bpm; P = 0.001) [25]. Łuczak et al. reported a significantly attenuated pre-to-peri-procedural HR rise (+3.2 vs +8.1 bpm; P = 0.009) [26]. Genç et al. found lower post-procedural HR following TRUS-Bx (71.84 ± 12.00 vs 78.15 ± 10.16 bpm; P = 0.05) [22]. Walker et al. found no significant between-group difference in vital signs during flexible cystoscopy [17].

Blood pressure was reported by three studies. Ketsuwan et al. demonstrated significantly lower post-procedural SBP and DBP in the VR group (SBP 145.4 vs 157.6 mmHg; DBP 94.3 vs 107.7 mmHg; both P = 0.001) [25]. Łuczak et al. reported a smaller pre-to-peri-procedural SBP rise with VR (+16.8 vs +22.8 mmHg; P = 0.029), with DBP non-significant [26]. Genç et al. found significantly lower post-procedural SBP (119.31 vs 129.09 mmHg; P = 0.01), with DBP non-significant [22]. Oxygen saturation remained within normal limits across all studies, with no clinically meaningful between-group differences. Weynants et al. reported VR patients tolerated significantly higher energy delivery during ESWL (55.2 vs 48.8 J; P = 0.037), an indirect marker of improved pain tolerance [30].

Adverse Effects

Adverse event data were reported by nine of eleven studies. No serious VR-related adverse events were identified across the entire evidence base, encompassing approximately 900 patients receiving VR. Mild VR-specific events were reported in three studies. Łuczak et al. reported moderate nausea and vertigo in a subset of VR patients during rigid cystoscopy, with significantly higher nausea scores in the VR group (1.8 ± 1.4 vs 1.1 ± 0.3; P = 0.001), the only outcome across the review in which VR performed significantly worse than standard care [26]. Weynants et al. reported claustrophobia in two of 82 VR participants (2.4%) during ESWL, without procedural interruption [30]. Gentile et al. recorded one instance of moderate motion sickness and one premature VR termination due to anxiety; data completion remained 97.6% [23]. Eight of nine reporting studies explicitly stated VR was well tolerated without procedural interruption. Bozkul et al. uniquely reported 24-hour post-procedural complications following TRUS-Bx. Urinary retention was significantly less frequent in the VR group (0% vs 14.3%; P = 0.027), with no differences in haematuria, dysuria, rectal bleeding, or infection. The authors noted this as an exploratory finding [21].

Certainty of Evidence

The certainty of evidence for each outcome was assessed using the GRADE framework [20], with downgrading considered for RoB, inconsistency, indirectness, imprecision, and publication bias. Certainty was rated as low for all outcomes, including procedural pain, procedural anxiety, patient satisfaction and comfort, physiological markers, and adverse events. The principal drivers of downgrading across these outcomes were substantial unexplained heterogeneity, instrument heterogeneity precluding formal quantitative pooling, small numbers of contributing studies, and unblinded outcome assessment in the absence of feasible participant or care-provider blinding. The certainty of evidence is summarised in Table 4.

**Table 4:** GRADE certainty of evidence assessment for included outcomes I²: Proportion of variability due to heterogeneity; GRADE: Grading of recommendations, assessment, development and evaluations; BP: Blood pressure; HR: Heart rate; NRS: Numerical rating scale; RCT: Randomized controlled trial; SMD: Standardised mean difference; SpO₂: Peripheral oxygen saturation; STAI: State-Trait Anxiety Inventory; TRUS-Bx: Transrectal ultrasound-guided prostate biopsy; VAS: Visual analogue scale; VR: Virtual reality

Outcome	Studies (participants)	Design	RoB	Inconsistency	Indirectness	Imprecision	Publication bias	Effect/finding	Certainty (GRADE)
Procedural pain (VAS/NRS 0–10; lower = less pain)	Six RCTs pooled (n = 709); seven RCTs reported pain (n = 875) [17,22,24-26,29,30]	RCTs	Serious	Serious	Serious	Not serious	Not assessed	Overall: SMD −0.68 (95% CI −1.25 to −0.11); P = 0.019; I² = 91.7%. Cystoscopy: SMD −0.16 (95% CI −0.32 to 0.00); I² = 0%. TRUS-Bx: SMD −1.84 (95% CI −2.82 to −0.85); I² = 82.5%. Favours VR overall.	Low
Procedural anxiety (STAI 20–80; lower = less anxiety)	Four RCTs (n = 496) [21,25,28,29]	RCTs	Serious	Very serious	Serious	Serious	Not assessed	SMD −1.39 (95% CI −2.64 to −0.14); P < 0.05; I² = 89% (Knapp-Hartung adjusted). Favours VR.	Low
Patient satisfaction/procedural comfort (higher = better)	Four RCTs contributing data (n = 410) (pooling not performed) [21,25,27,29]	RCTs	Serious	Not serious	Serious	Serious	Not assessed	Narrative synthesis only. Ketsuwan: VAS-sat 9.4 vs 8.8; P = 0.001. Toraman: PPHEN 65.4 vs 42.4; P < 0.001. Bozkul: VAS-comfort 9.4 vs 7.5; P < 0.001. Robinson: VAS-sat non-significant (P = 0.520).	Low
Physiological markers (HR, BP, SpO₂)	Five RCTs (n = 639) (pooling not performed) [17,22,24-26]	RCTs	Serious	Serious	Serious	Not serious	Not assessed	Narrative synthesis only. Heart rate: 4/5 studies favour VR significantly. Systolic BP: 3/4 studies favour VR significantly. SpO₂ / respiratory rate: one study favours VR. Walker (flexible cystoscopy): all markers non-significant.	Low
Adverse events (VR-related)	Nine RCTs reporting adverse events data (approximate n = 900 VR patients) [17,21,22-27,29,30]	RCTs	Serious	Not serious	Not serious	Serious	Not assessed	No serious VR-related adverse events identified across the evidence base. Mild events only: nausea/vertigo (Łuczak); claustrophobia in two of 82 patients (Weynants); motion sickness in one patient (Gentile). Two studies did not report adverse event data (Genç, Sibal).	Low

Discussion

Principal Findings

This systematic review and meta-analysis synthesised evidence from 11 RCTs encompassing 1,557 participants across eight countries, evaluating HMD VR as a non-pharmacological adjunct during awake urological procedures. Pooled analyses showed a statistically significant reduction in procedural pain (SMD −0.68, 95% CI −1.25 to −0.11; P = 0.019) and procedural anxiety (SMD −1.39, 95% CI −2.64 to −0.14; P < 0.05) favouring VR, alongside narrative improvements in patient satisfaction and physiological stress markers, with no serious VR-related adverse events. Subgroup analysis revealed marked divergence in pain response by procedure type; VR produced negligible benefit during cystoscopy (SMD −0.16) but substantial reductions during TRUS-Bx (SMD −1.84), indicating that VR's effects are not uniform across awake urological procedures and are likely modulated by the intensity of the underlying nociceptive and psychological stimulus.

Comparison With Existing Evidence

These findings are biologically plausible and consistent with three decades of procedural pain research. The gate control theory of pain holds that non-nociceptive sensory input attenuates upward transmission of pain signals at the spinal level, while attentional load theory proposes that finite cognitive bandwidth allocated to immersive environments limits the conscious processing of pain and anxiety [6-8]. Functional MRI evidence suggests these mechanisms have a neurobiological basis, with VR distraction reducing pain-evoked activity in cortical and subcortical regions involved in pain processing [31]. These findings are broadly consistent with a substantial cross-specialty literature. The most authoritative cross-procedure synthesis to date reported a pooled SMD of −0.78 across 92 RCTs in BMC Medicine [8]; while not directly comparable to the present urological estimate given differences in procedures and populations, it indicates a similar overall direction of effect. Comparable benefits have been reported in interventional cardiology, gastrointestinal endoscopy, burn wound debridement, and labour analgesia, with the largest effects generally observed during procedures combining sustained nociceptive stimulation with high anticipatory anxiety [6,9].

Interpretation by Procedure

The most striking finding of the present analysis is the marked divergence in VR analgesic efficacy between procedure types, with the formal test for subgroup differences strongly significant (P = 0.001). The most parsimonious explanation lies in differential procedural intensity. Cystoscopy, particularly when flexible, lubricated with intraurethral lidocaine, and competently performed, typically generates moderate baseline pain, with mean VAS scores in the 3-5 range across both intervention and control arms of the included trials. This may leave limited 'analgesic headroom' for any non-pharmacological intervention to demonstrate an effect, and even intraurethral lidocaine, the established pharmacological standard of care, produced only an SMD of −0.22 in a 2019 meta-analysis of 1,549 patients [32]. The cystoscopy subgroup estimate of the present review (SMD −0.16) is virtually identical to that of Lopes et al. (SMD −0.16) and Yao et al. (SMD −0.28), three independent analyses converging on the same conclusion that VR offers, at best, a marginal pain benefit during cystoscopy [11,33]. The TRUS-Bx, by contrast, presents a fundamentally different procedural and psychological stimulus: it is longer, involves both transrectal probe insertion and multiple percutaneous needle cores, and is overlaid with substantial cancer-related anticipatory anxiety. Reported control-arm pain scores in the included TRUS-Bx trials were correspondingly higher (mean VAS 5.0-6.8), providing the necessary headroom for VR to demonstrate measurable benefit. A similar pattern of effect-size scaling with procedural intensity has been reported across endoscopy, where colonoscopy (SMD −0.70) and laparoscopy (SMD −0.54) yielded substantially larger VR effects than cystoscopy [33].

Several caveats nonetheless temper interpretation of the TRUS-Bx finding. The pooled estimate is based on only two studies, both conducted in Turkish nursing-led centres and rated at 'some concerns' or 'high' overall risk of bias [22,29]. Genç et al. assessed pain by patient recall 15 minutes after the procedure, a methodological concern previously identified as the principal driver of heterogeneity in endoscopy meta-analyses, with retrospective recall systematically inflating effect sizes compared to real-time intraprocedural assessment [22,33]. Within-subgroup heterogeneity for the TRUS-Bx estimate was substantial (I² = 82.5%), and the very large SMD should be interpreted as exploratory and hypothesis-generating rather than confirmatory. Adequately powered, prospectively registered, methodologically rigorous TRUS-Bx RCTs incorporating real-time pain assessment and outcome assessor blinding are needed before the magnitude of VR's benefit in this setting can be considered established.

Methodological Context

Beyond the substantive findings, this review should be situated within the rapidly evolving methodological landscape of urological evidence synthesis. The earlier narrative mini-review by Perucchini et al. provided a valuable conceptual foundation but did not undertake formal risk of bias assessment or certainty-of-evidence appraisal [10]. Recent British Journal of Urology International guidance on systematic reviews and meta-analyses in urology has cautioned that quantitative synthesis without critical interpretation risks producing 'cookie-cutter' reviews, methodologically formulaic but interpretively insubstantial [34]. The present analysis was designed in awareness of this concern: every pooled estimate is qualified by within-procedure heterogeneity, prediction intervals, GRADE certainty ratings [20], and explicit acknowledgement of where the evidence base is small, underpowered, or methodologically vulnerable. In a literature where 11 RCTs have been conducted across five different procedure types with no two studies replicating another in procedure, population, and outcome instrument, the meaningful contribution of a systematic review is arguably as much in clarifying what cannot yet be concluded as in pooling what can.

Clinical Implications

The clinical implications are pragmatic and procedure-specific. The most defensible inference is that VR represents a credible non-pharmacological adjunct for the urological procedures characterised by the highest baseline distress, particularly TRUS-Bx and ESWL, and is unlikely to deliver meaningful benefit during routine flexible cystoscopy. Although it concerns a different intervention, this selective implementation framing parallels how non-pharmacological adjuncts have been positioned previously in urology: the European Association of Urology (EAU) Section of Uro-Technology systematic review on intraprocedural music reached a similar conclusion that procedural distraction is most beneficial where baseline distress is greatest and recommended targeted rather than universal adoption [35]. The safety profile across approximately 900 patients, with no serious adverse events, suggests the practical barriers to selective implementation may be modest, although implementation costs and training requirements were not evaluated in this review. The exploratory finding from Bozkul et al. of reduced post-biopsy urinary retention in VR recipients, if reproduced in adequately powered trials, might extend VR's potential value beyond patient experience into measurable clinical outcomes [21].

Strengths and Limitations

Several limitations warrant explicit acknowledgement. The pooled estimates are based on a small number of contributing trials, with some outcome subgroups including as few as two studies, and substantial unexplained heterogeneity was observed across both pain (I² = 91.7%) and anxiety (I² = 89%) analyses. Three of the 11 RCTs were rated at high risk of bias, and the inability to blind participants and care providers represents a systematic source of performance bias across the entire evidence base; only one included study employed outcome assessor blinding. Methodological heterogeneity between trials in VR hardware, content type, delivery timing, and outcome instruments could not be adequately interrogated through subgroup or meta-regression analysis given the limited study numbers. Five of the 11 trials originated from Turkish nursing-led centres, which may limit generalisability. Ten of 11 studies enrolled exclusively or predominantly male participants, leaving the effectiveness of VR during female cystoscopy and other female-predominant urological procedures inadequately characterised. Sensitivity analyses using alternative between-study variance estimators, excluding studies at high risk of bias, or excluding studies using delayed pain recall were not undertaken, given the small number of contributing studies per outcome; the pooled estimates should therefore be regarded as provisional.

The search was conducted to the pre-specified cut-off of April 2026 registered in the review protocol. Randomised trials published after this date, including further studies of VR during urodynamic testing, extracorporeal shock wave lithotripsy, and flexible cystoscopy, were therefore not eligible for inclusion and may refine the pooled estimates; an updated synthesis incorporating these trials will be warranted as the evidence base matures. None of the included trials examined longer-term outcomes such as compliance with surveillance schedules, willingness to undergo repeat procedures over time, or cost-effectiveness.

## Conclusions

This systematic review and meta-analysis of 11 RCTs suggests that head-mounted display VR may reduce procedural pain and anxiety during selected awake urological procedures, although substantial heterogeneity and low certainty of evidence mean the pooled estimates should be interpreted with caution. No serious VR-related adverse events were identified across approximately 900 patients receiving the intervention. The benefit appears procedure-specific rather than uniform, with larger reductions observed during higher-intensity procedures such as TRUS-Bx and ESWL but negligible effect during routine flexible cystoscopy; however, the TRUS-Bx and ESWL findings rest on very few studies and should be considered exploratory. From a clinical standpoint, these findings support targeted rather than universal implementation, prioritising procedures characterised by sustained nociceptive stimulation and high anticipatory distress. Adequately powered, methodologically rigorous trials, particularly in TRUS-Bx, incorporating outcome assessor blinding, real-time intraprocedural pain assessment, standardised reporting of VR hardware and content, and pre-specified clinical endpoints beyond patient-reported experience are needed to establish whether the apparent benefit is reproducible and to inform routine practice. Given the heterogeneity, methodological limitations, and small number of contributing trials, these findings should be regarded as preliminary.
